# The Value of Deep Learning Image Reconstruction in Improving the Quality of Low-Dose Chest CT Images

**DOI:** 10.3390/diagnostics12102560

**Published:** 2022-10-21

**Authors:** Jiu-Ming Jiang, Lei Miao, Xin Liang, Zhuo-Heng Liu, Li Zhang, Meng Li

**Affiliations:** 1 Department of Diagnostic Radiology, National Cancer Center/National Clinical Research Center for Cancer/Cancer Hospital, Chinese Academy of Medical Sciences and Peking Union Medical College, Beijing 100021, China; 2 Medical Statistics Office, National Cancer Center/National Clinical Research Center for Cancer/Cancer Hospital, Chinese Academy of Medical Sciences and Peking Union Medical College, Beijing 100021, China; 3 CT Research Center, GE Healthcare China, Shanghai 200131, China

**Keywords:** deep learning, low-dose computed tomography, image quality, lung

## Abstract

This study aimed to evaluate the value of the deep learning image reconstruction (DLIR) algorithm (GE Healthcare’s TrueFidelity™) in improving the image quality of low-dose computed tomography (LDCT) of the chest. First, we retrospectively extracted raw data of chest LDCT from 50 patients and reconstructed them by using model-based adaptive statistical iterative reconstruction-Veo at 50% (ASIR-V 50%) and DLIR at medium and high strengths (DLIR-M and DLIR-H). Three sets of images were obtained. Next, two radiographers measured the mean CT value/image signal and standard deviation (SD) in Hounsfield units at the region of interest (ROI) and calculated the signal-to-noise ratio (SNR) and contrast-to-noise ratio (CNR). Two radiologists subjectively evaluated the image quality using a 5-point Likert scale. The differences between the groups of data were analyzed through a repeated measures ANOVA or the Friedman test. Last, our result show that the three reconstructions did not differ significantly in signal (*p* > 0.05) but had significant differences in noise, SNR, and CNR (*p* < 0.001). The subjective scores significantly differed among the three reconstruction modalities in soft tissue (*p* < 0.001) but not in lung tissue (*p* > 0.05). DLIR-H had the best noise reduction ability and improved SNR and CNR without distorting the image texture, followed by DLIR-M and ASIR-V 50%. In summary, DLIR can provide a higher image quality at the same dose, enhancing the physicians’ diagnostic confidence and improving the diagnostic efficacy of LDCT for lung cancer screening.

## 1. Introduction

Lung cancer has the second highest incidence rate and the highest mortality rate among all malignancies [1], and an early detection and clinical intervention are essential for improving the patient’s prognosis. Since most early stage lung cancers are asymptomatic, screening for early stage lung cancer to improve the patient’s prognosis has always been a focus of lung cancer research. Low-dose computed tomography (LDCT) of the chest is the only effective lung cancer screening method at present and can significantly reduce lung cancer mortality in high-risk groups [2,3]. Chest LDCT is suitable for large-scale population screening. This method mainly reduces the dose by reducing the intensity of the tube current (mAs), but the dose reduction also leads to an increase in noise, which affects the image quality and diagnostic performance [4,5].

The CT reconstruction algorithm is a critical factor affecting image quality. The traditional filtered back projection (FBP) algorithm can obtain high-quality images from high-dose scanning. However, after the dose is reduced, the image noise increases significantly, affecting the observation and judgment of low-contrast structures and lesions [6,7]. Therefore, various iterative reconstruction (IR) algorithms are currently used in the processing of chest LDCT data to reduce image noise and artifacts. However, it has been found that regardless of the IR technique applied, the smooth artifacts at the tissue boundaries tend to cause a “waxy” and “plastic-like” speckled image appearance, especially with high-intensity IR [8,9], which is considered to be a technical flaw in IR.

In recent years, with the explosive development of artificial intelligence (AI) in the medical field, a deep learning image reconstruction (DLIR) method based on a deep neural network (DNN) was proposed to reduce image noise and improve spatial resolution [10,11], and it is expected to be superior to the existing IR techniques in image quality, dose performance, and reconstruction speed. To our knowledge, there are only two commercially available CT image reconstruction algorithms using the DLIR method that have been approved by the FDA: TrueFidelity^TM^ by GE Healthcare and AiCE by Canon Medical Systems. In our study, the DLIR technology used was GE Healthcare’s TrueFidelity™. At present, there are few reports on the application of DLIR in chest LDCT, and its effect has yet to be evaluated. The purpose of this study was to reconstruct the raw data of chest LDCT by using the DLIR algorithm (TrueFidelity^TM^) and model-based adaptive statistical iterative reconstruction-Veo (ASIR-V) on a GE Healthcare CT system, compare the image quality between the two reconstruction algorithms, and discuss the application value of DLIR in improving the chest LDCT image quality.

## 2. Materials and Methods

### 2.1. Study Population

Ethical approval was obtained from the Institutional Review Board of the Cancer Hospital Chinese Academy of Medical Sciences (protocol number NCC3609, approval date 2 August 2022) for this retrospective analysis, and the requirement to obtain informed consent was waived. The imaging data of 50 patients at the Cancer Hospital of the Chinese Academy of Medical Sciences from September to October 2021 were collected. The inclusion criteria included patients who underwent chest LDCT scans at Revolution CT for cancer prevention screening or periodic lung nodule review. The exclusion criteria included patients with severe respiratory diseases and images of poor quality that could not be accurately measured.

### 2.2. Image Acquisition

All patients were scanned with a Revolution CT from GE Healthcare. The patient was in a supine position in the middle of the examination bed with the head advanced, arms elevated, and hands behind the head. To ensure an adequate image quality, any metal jewelry or clothing components on the patient’s neck and chest were removed before scanning, and standardized breathing training was executed to reduce the breathing motion artifacts. The scanning method was as follows: spiral scanning; the scanning area: from the apex to the base of the lung; and the scanning parameters: a tube voltage of 120 kV, an automatic tube current modulation of 20~30 mAs, a noise index of 17, a pitch of 0.992:1, and a rotation time of 0.5 s.

### 2.3. Algorithm Training and Image Reconstruction

The DLIR technology used in this study was GE Healthcare’s TrueFidelity™. This technique utilizes high-quality projection data obtained by FBP at high doses as an ideal model to obtain a large number of real image sets from laboratory and clinical environments to train and verify the DNN. First, the low-dose sinogram was input to the DNN, and then the DNN output image was compared with the corresponding high-dose FBP output image. The difference between the low-dose output image and the high-dose output image was found, and these features were fed back into the DNN. The DNN tends to make the low-dose image the ideal model by enhancing (or weakening) some equations. This process was repeated until the model tended to be stable, and finally, the accuracy and stability of this model were verified with new clinical or virtual cases [12]. Eventually, the DLIR engine is trained to create TrueFidelity CT images similar to high-dose FBP at low doses. In addition, DLIR provides three reconstruction strength levels (low, medium, and high) to control the amount of noise reduction [12,13].

The raw LDCT scanning data of all the patients were reconstructed by ASIR-V at a level of 50% (ASIR-V 50%) and DLIR at medium and high strengths (DLIR-M and DLIR-H) to obtain three sets of images with a layer thickness of 1.25 mm. All images were transmitted to the GE AW4.7 workstation, and the image information was anonymized.

### 2.4. Radiation Dose

To assess the radiation exposure, the volume CT dose index (CTDI_vol_) and dose-length-product (DLP) were reviewed from the electronically logged protocol for each LDCT acquisition. The effective radiation dose of chest CT was calculated by multiplying the DLP by the region-specific conversion coefficient k of 0.014 mSv/mGy cm [14].

### 2.5. Objective Image Analysis

Two radiographers with 3 years of experience in radiology were responsible for measuring the mean CT value and standard deviation (SD) in Hounsfield units (HU) at the region of interest (ROI). The mean CT value represents the signal of the image. The noise was defined as the SD of the mean CT value in the same ROI [14]. Subsequently, the signal-to-noise ratio (SNR) and contrast-to-noise ratio (CNR) were calculated [15]. The ROI was set as follows: each ROI area was approximately 100 mm^2^; ROI_aorta_ was placed in the center of the descending aorta, avoiding the tube wall; ROI_lung_ was placed in the normal right interlobar fissure with few lung markings; ROI_muscle_ was placed in the central area with uniform density in the right subscapular muscle; ROI_liver_ was placed in the S7 segment of the right lobe of the liver; and ROI_vertebrae_ was placed at the central 12th thoracic vertebrae. When setting the ROI, the blood vessels, bile duct, and lesion tissue were avoided. Background noise was evaluated based on air located 3–5 cm in front of the sternum. The SNR and CNR were calculated as follows: $SNR=\frac{HU_{ROI}}{SD_{ROI}}$; $CNR=\frac{2{\left(HU_{Target}-HU_{Background\;air}\right)}^{2}}{SD_{Target}{}^{2}+SD_{Background\;air}{}^{2}}$.

### 2.6. Subjective Image Analysis

Two radiologists with 3 and 10 years of experience performed a subjective analysis of the three groups of images. The radiologists were unaware of the image reconstruction techniques and patient characteristics. The images are displayed in a random order in a preset window, displaying a sequence at a time. The radiologists could scroll through the image and adjust the window width and window position at random. We used a 5-point scale to evaluate the subjective image quality of soft tissue and lung tissue. The scoring standard for soft tissue was as follows: 1 = poor mediastinal contrast, unacceptable image; 2 = slightly poor mediastinal contrast, suboptimal image; 3 = moderate mediastinal contrast, acceptable image; 4 = good mediastinal contrast, good image; and 5 = excellent mediastinal contrast, optimal image. The scoring standard for the lung tissue was as follows: 1 = unclearly displayed lung markings, unacceptable image; 2 = fussy display of lung markings, suboptimal image; 3 = generally displayed lung, acceptable image; 4 = clearly displayed lung markings, good image; and 5 = lung excellent display of markings, optimal image.

### 2.7. Statistical Analysis

IBM SPSS Statistics for Windows, version 26.0 (Armonk, NY, USA) was used for the statistical analysis. The objective data following a normal distribution are presented as the mean ± SD, while nonnormally distributed data are presented as the median (interquartile range, IQR). The differences between the groups of data were analyzed through a repeated measures ANOVA or the Friedman test, depending on the normality. Bonferroni multiple comparisons were used for pairwise comparisons between any two groups. The subjective data are presented as the median (IQR) and the differences between the groups were analyzed through the Friedman test. A *p* value < 0.05 was considered statistically significant.

Intraclass correlation coefficients (ICCs) were calculated to evaluate an agreement on the objective data between the two radiographers. An ICC value >0.75 indicates a good consistency; 0.4~0.75, a general consistency; and <0.4, a poor consistency. Cohen’s kappa statistic was calculated for an agreement on the independent scoring of the image quality between the two radiologists. A kappa statistic of 0.81~1.00 implies an excellent agreement; 0.61~0.80, a substantial agreement; 0.41~0.60, a moderate agreement; 0.21~0.40, a fair agreement; and 0.00~0.20, a poor agreement.

## 3. Results

According to the exclusion criteria for inclusion, the CT images of 48 patients were finally selected. Among them, there were 22 males [mean age ± standard deviation (SD) 51.45 ± 9.57 years] and 26 females (mean age ± SD 52.31 ± 8.94 years).

### 3.1. Radiation Dose

The mean CTDI_vol_, DLP, and effective dose of the 48 patients were 2.04 ± 0 mGy, 79.69 ± 4.81 mGy*cm, and 1.07 ± 0.07 mSv, respectively.

### 3.2. Objective Analysis

**Figure 1:**
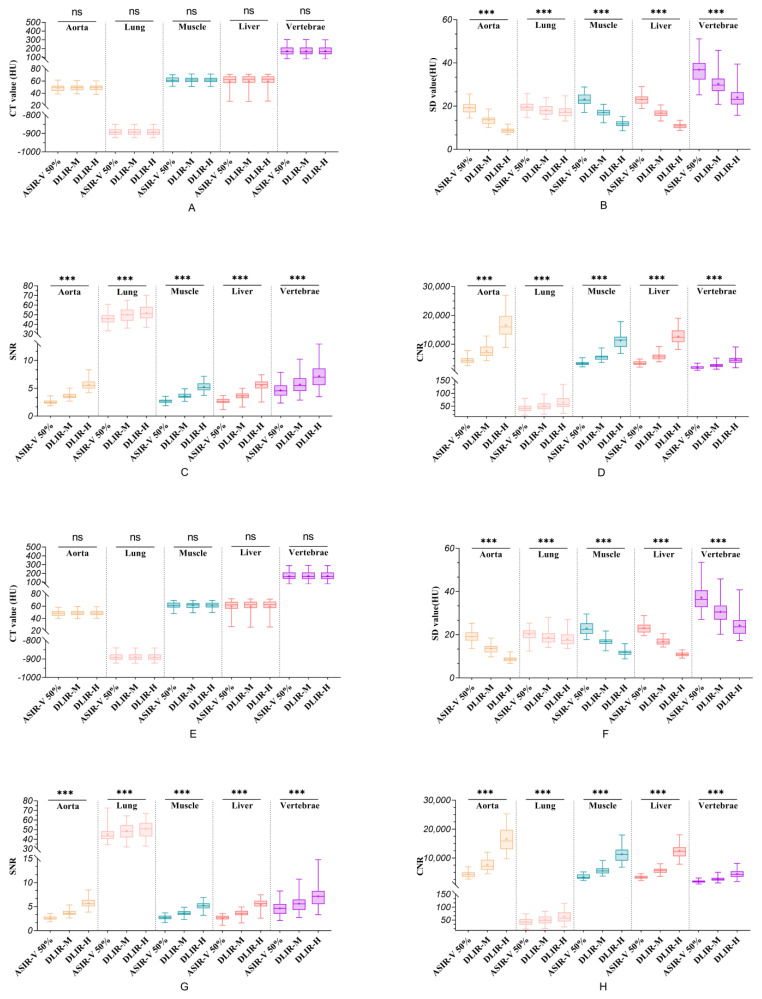
Boxplots of all quantitative data. (**A**−**D**) show the CT value, SD value, SNR, and CNR measured by Reader 1, respectively. (**E**−**H**) show the CT value, SD value, SNR, and CNR measured by Reader 2, respectively. ns indicates that the difference between the three groups is not statistically significant (*p* > 0.05). *** indicates that the difference between the three groups is statistically significant (*p* < 0.001). + in the boxplot indicates the mean value.

The mean CT values measured by the two radiographers were consistent (ICC value: 0.921~0.995). There were no significant differences in the mean CT values among the three image reconstruction methods for the aorta, lung, muscle, liver, and vertebrae (all *p* > 0.05), i.e., the reconstruction method had no effect on the signal intensity of the images. The results are presented in Table 1 and Figure 1A,E.

The SD values measured by the two radiographers in the lung were generally consistent (ICC value: 0.716~0.815), and the SD values in the aorta, muscle, liver, and vertebrae were consistent (ICC value: 0.817~0.937). There were significant differences in the SD values among the three image reconstruction methods for the aorta, lung, muscle, liver, and vertebrae (all *p* < 0.05), i.e., the reconstruction method had a significant effect on the image noise. DLIR-H had the lowest noise in the ROIs of the three constructed images, followed by DLIR-M and ASIR-V 50%. Compared with ASIR-V 50%, DLIR-H reduced the noise by approximately 54.74%, 52.88%, 48.35%, 35.90%, and 11.55% in the aorta, liver, muscle, vertebrae, and lung tissue, respectively. A pairwise comparison between the groups showed that the three reconstruction modalities showed significant differences in the aorta, lung tissue, subscapularis muscle, liver, and vertebrae (all *p* < 0.05). The results are presented in Table 2 and Figure 1B,F.

The SNR and CNR calculated by the two radiographers were consistent (ICC value: 0.739~0.976). There were significant differences in the SNR and CNR among the three image reconstruction methods for the aorta, lung, muscle, liver, and vertebrae (all *p* < 0.05). DLIR-H had the highest SNR and CNR in each ROI, and ASIR-V 50% had the lowest. Compared with ASIR-V 50%, DLIR-H improved the SNR by approximately 125.35%, 112.40%, 93.75%, 53.95%, and 15.65% and enhanced the CNR by approximately 276.55%, 274.50%, 251.95%, 147.65%, and 35.10% in the aorta, liver, muscle, vertebrae, and lung tissue, respectively. Pairwise comparisons between the groups showed statistically significant differences among all methods (*p* < 0.05). The results are presented in Table 3 and Table 4 and Figure 1C,D,G,H.

### 3.3. Subjective Analysis

**Figure 2:**
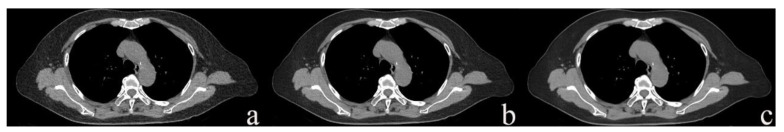
Soft tissue images of a 62-year-old female after chest LDCT. Three reconstruction methods were used: ASIR-V 50% (**a**), DLIR-M (**b**), and DLIR-H (**c**). The image signal did not significantly vary across different reconstructions (all *p* > 0.05). Image noise significantly varied across different reconstructions (all *p* < 0.05). Both radiologists agreed that DLIR-H had the best image quality, with low noise and a natural texture.

**Figure 3:**
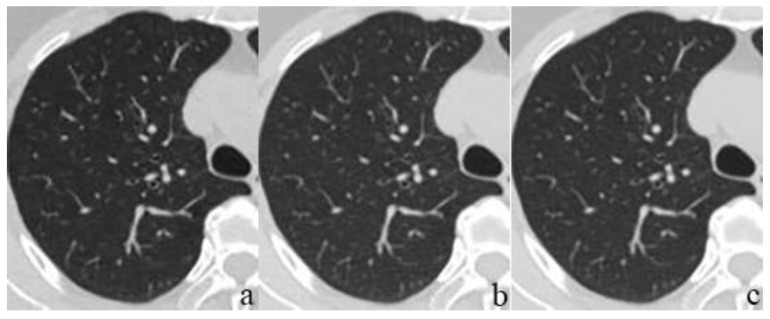
Lung tissue images of a 62-year-old female after chest LDCT. Three reconstruction methods were used: ASIR-V 50% (**a**), DLIR-M (**b**), and DLIR-H (**c**). The image signal did not significantly vary across different reconstructions (all *p* > 0.05). Image noise slightly varied across different reconstructions (all *p* < 0.05). However, both radiologists agreed that DLIR did not visualize the lung tissue more clearly.

The subjective analysis results show significant differences in the image quality for soft tissue among the three reconstruction methods (all *p* < 0.05). The image score of DLIR was higher than that of ASIR-V 50%. However, the difference in the image quality for the lung tissue was not statistically significant among the three reconstruction methods (*p* = 0.121 and 0.069). Both radiologists believed that DLIR had an outstanding noise reduction ability in soft tissue. DLIR-H had the best image quality, with a low noise and a natural texture (Figure 2). However, DLIR did not visualize the lung tissue more clearly (Figure 3). The subjective scores between the two radiologists were consistent (kappa value range: 0.48–0.91). The results are presented in Table 5.

## 4. Discussion

Different reconstruction algorithms lead to different image qualities. In view of the wide application of chest LDCT, in this study, we used both objective and subjective evaluation methods to compare the effect of the DLIR algorithm and ASIR-V algorithm on the image quality of chest LDCT. However, we should be aware that there is not an official definition of the terms low dose or standard dose [16]. Since expectations concerning the image quality can substantially differ between institutions, the definition of a high or low dose will vary. The radiation dose in our study is in line with the current guideline recommendation [17]. In this study, the objective parameters of image quality were measured by two radiographers, and there was a good agreement between the measured values. The objective analysis results showed that the reconstruction method had no effect on the signal intensity of the images. However, DLIR had a significant advantage in reducing the image noise and improving the SNR and CNR. The noise reduction abilities of the three reconstruction methods from best to worst were DLIR-H, DLIR-M, and ASIR-V 50%. Compared with the ASIR-V 50% algorithm, DLIR-H was able to reduce noise in the aorta, liver, muscle, vertebrae, and lung tissue by approximately 54.74%, 52.88%, 48.35%, 35.90%, and 11.55%, respectively, maintaining a high resolution while significantly reducing noise and artifacts. The subjective evaluation results showed that DLIR significantly improved the image quality for the soft tissue and maintained the real noise texture. The image quality with DLIR was better than that with the standard algorithm ASIR-V 50%, but there was no significant difference in the visual effect among the three reconstruction methods when observing the lung tissue. Consistent with previous results [18,19], this study further confirmed that the DLIR reconstruction algorithm could significantly improve the quality of chest LDCT images.

Noise is a key factor affecting the image quality. A large amount of noise will directly affect the density resolution of the image and reduce or even mask the visibility of some features, especially low-contrast structures. ASIR-V is a hybrid reconstruction technique that combines the model-based iterative advantages of ASIR with a real-time reconstruction and model-based iterative reconstruction (MBIR) [20]. ASIR-V successfully reduces the radiation dose of chest LDCT and provides a relatively good image quality; thus, this method is now widely used in clinical practice [21,22]. However, this is a concession to the low-dose image standard established after a comprehensive consideration of the existing technology and doses. Research shows that radiologists are most satisfied with the images reconstructed by the FBP algorithm under high-dose conditions. The advantages of the ASIR-V algorithm are based on the complexity of the model. A limited degree of reconstruction increasingly affects the image texture as the iterative intensity increases, resulting in an unnatural visual effect [8,9]. The True Fidelity™ engine is based on the raw data acquired with a CT scanner and directly uses FBP-reconstructed images under ideal conditions as the training standard to improve the IR algorithm to reduce the degree to which noise will distort the image texture [12]. In this study, the chest LDCT images reconstructed with DLIR-H did not exhibit the oversmoothing phenomenon, but it showed the robust noise reduction ability of the algorithm under low-dose scanning conditions and maintained a more realistic image texture. Recent studies on DLIR have also proven that it can improve the image quality and reduce the radiation dose in abdominal CT examinations, chest angiography in children, CT urography, etc. [23,24,25,26].

Among the different tissues and organs (aorta, lung tissue, liver, muscle, and vertebrae) on chest LDCT images, DLIR had the best noise reduction ability for soft tissue density organs or tissues (aorta, liver, and muscle), followed by bone and lung. LDCT is widely used for lung cancer screening because the lungs are rich in gas, have a naturally good contrast, and are more tolerant to noise than other anatomical structures (abdomen or head). We suggest that this may be a reason why the visual effect improvement of DLIR is evident in soft tissue but not in lung tissue. On the other hand, our subjective evaluation is based on normal lung tissue rather than pathological details. This may also account for the lack of difference in the subjective evaluation. In addition to lung cancer screening, chest LDCT can also screen and diagnose some common major chronic noncommunicable diseases, such as coronary artery calcification, fatty liver, osteoporosis, and extrapulmonary (including mediastinum, thyroid, breast, and upper abdominal organs) tumor lesions. DLIRs are more effective in reducing the noise of soft tissue and bone and improving the image contrast, which is beneficial for the detection of major chronic noninfectious diseases, in addition to lung cancer, by thoracic LDCT, and is important for improving the cost-effectiveness and significance of thoracic LDCT for lung cancer screening.

Our study still has certain limitations. First, this study only compared normal anatomical tissues with standard reconstruction algorithms and did not explore the detection capability of the algorithms for the lesions. In further studies, the detection capabilities of the two different reconstruction methods will be validated for different disease types. Second, the sample size of this study was relatively small and the study was conducted in a single center. Larger sample sizes need to be included to further validate the current research results.

In summary, for LDCT, DLIR can provide a higher image quality at the same dose, enhancing the physicians’ diagnostic confidence and improving the diagnostic efficacy of LDCT for lung cancer screening.

## Figures and Tables

**Table 1:** Comparison of image CT values among the three groups.

CT Value (HU)	ASIR-V 50%	DLIR-M	DLIR-H	*p*	*p*		
					ASIR-V 50% vs. DLIR-M	ASIR-V 50% vs. DLIR-H	DLIR-M vs. DLIR-H
Aorta							
ICC	0.948	0.951	0.955				
Reader 1	48.64 ± 5.47	48.76 ± 5.35	48.72 ± 5.36	0.515 †	1.000	1.000	1.000
Reader 2	48.64 ± 5.04	48.79 ± 4.99	48.78 ± 4.91	0.394 †	0.891	1.000	1.000
Lung							
ICC	0.960	0.958	0.961				
Reader 1	−891.66 ± 18.06	−891.65 ± 17.82	−891.63 ± 17.88	0.866 †	1.000	1.000	1.000
Reader 2	−889.88 ± 19.30	−890.00 ± 19.12	−889.88 ± 19.14	0.622 †	1.000	1.000	1.000
Muscle							
ICC	0.926	0.921	0.925				
Reader 1	61.55 ± 4.65	61.67 ± 4.90	61.66 ± 4.83	0.681 †	1.000	1.000	1.000
Reader 2	61.27 ± 4.81	61.21 ± 4.94	61.28 ± 4.83	0.804 †	1.000	1.000	0.780
Liver							
ICC	0.987	0.990	0.992				
Reader 1	62.40 (10.82)	62.90 (10.45)	63.05 (10.03)	0.717 ‡	1.000	1.000	1.000
Reader 2	62.13 (11.08)	62.46 (10.27)	62.41 (10.20)	0.763 ‡	1.000	1.000	1.000
Vertebrae							
ICC	0.993	0.994	0.995				
Reader 1	170.50 ± 52.68	170.69 ± 53.25	170.53 ± 53.03	0.662 †	1.000	1.000	0.790
Reader 2	170.15 ± 52.04	170.32 ± 52.91	170.23 ± 52.77	0.750 †	1.000	1.000	1.000

Note: HU = Hounsfield units. ICC = intraclass correlation coefficient. ASIR-V 50% = adaptive statistical iterative reconstruction-Veo at a level of 50%; DLIR-M and DLIR-H = deep learning image reconstruction in medium and high strengths, respectively. † indicates normally distributed data compared using repeated measures ANOVA. ‡ indicates nonnormally distributed data compared with the Friedman test.

**Table 2:** Comparison of image noise in the three groups.

Noise (HU)	ASIR-V 50%	DLIR-M	DLIR-H	*p*	*p*		
					ASIR-V 50% vs. DLIR-M	ASIR-V 50% vs. DLIR-H	DLIR-M vs. DLIR-H
Aorta							
ICC	0.921	0.934	0.932				
Reader 1	19.35 ± 2.70	13.58 ± 1.96	8.74 ± 1.22	<0.001 †	<0.001	<0.001	<0.001
Reader 2	19.16 ± 2.66	13.42 ± 1.96	8.69 ± 1.20	<0.001 †	<0.001	<0.001	<0.001
Lung							
ICC	0.815	0.716	0.751				
Reader 1	19.79 ± 2.59	18.18 ± 2.44	17.45 ± 2.51	<0.001 †	<0.001	<0.001	0.001
Reader 2	20.22 ± 2.93	18.75 ± 3.04	17.94 ± 2.93	<0.001 †	<0.001	<0.001	<0.001
Muscle							
ICC	0.902	0.851	0.817				
Reader 1	23.13 ± 2.93	17.10 ± 1.80	11.89 ± 1.44	<0.001 †	<0.001	<0.001	<0.001
Reader 2	22.87 ± 3.08	17.01 ± 2.09	11.87 ± 1.60	<0.001 †	<0.001	<0.001	<0.001
Liver							
ICC	0.906	0.862	0.834				
Reader 1	23.07 ± 2.35	16.81 ± 1.75	10.86 ± 1.08	<0.001 †	<0.001	<0.001	<0.001
Reader 2	23.13 ± 2.21	16.86 ± 1.61	10.91 ± 0.99	<0.001 †	<0.001	<0.001	<0.001
Vertebrae							
ICC	0.937	0.896	0.896				
Reader 1	36.82 (7.66)	29.72 (5.69)	23.16 (5.68)	<0.001 ‡	<0.001	<0.001	<0.001
Reader 2	36.28 (7.81)	30.50 (6.47)	23.69 (6.31)	<0.001 ‡	<0.001	<0.001	<0.001

Note: HU = Hounsfield units. ICC = intraclass correlation coefficient. ASIR-V 50% = adaptive statistical iterative reconstruction-Veo at a level of 50%; DLIR-M and DLIR-H = deep learning image reconstruction in medium and high strengths, respectively. † indicates normally distributed data compared using repeated measures ANOVA. ‡ indicates nonnormally distributed data compared with the Friedman test.

**Table 3:** Comparison of SNR among the three groups.

SNR	ASIR-V 50%	DLIR-M	DLIR-H	*p*	*p*		
					ASIR-V 50% vs. DLIR-M	ASIR-V 50% vs. DLIR-H	DLIR-M vs. DLIR-H
Aorta							
ICC	0.911	0.913	0.925				
Reader 1	2.46 (0.53)	3.50 (0.72)	5.51 (1.24)	<0.001 ‡	<0.001	<0.001	<0.001
Reader 2	2.47 (0.59)	3.56 (0.74)	5.60 (1.29)	<0.001 ‡	<0.001	<0.001	<0.001
Lung							
ICC	0.757	0.739	0.790				
Reader 1	45.80 ± 6.03	49.95 ± 7.01	52.12 ± 7.54	<0.001 †	<0.001	<0.001	<0.001
Reader 2	43.37 (7.94)	48.05 (12.79)	50.95 (13.96)	<0.001 ‡	0.018	<0.001	<0.001
Muscle							
ICC	0.893	0.837	0.817				
Reader 1	2.70 ± 0.38	3.64 ± 0.47	5.26 ± 0.77	<0.001 †	<0.001	<0.001	<0.001
Reader 2	2.73 ± 0.43	3.65 ± 0.55	5.26 ± 0.85	<0.001 †	<0.001	<0.001	<0.001
Liver							
ICC	0.976	0.965	0.961				
Reader 1	2.65 (0.68)	3.65 (0.83)	5.67 (1.22)	<0.001 ‡	<0.001	<0.001	<0.001
Reader 2	2.68 (0.71)	3.59 (0.89)	5.65 (1.18)	<0.001 ‡	<0.001	<0.001	<0.001
Vertebrae							
ICC	0.971	0.966	0.943				
Reader 1	4.66 ± 1.32	5.67 ± 1.65	7.18 ± 2.09	<0.001 †	<0.001	<0.001	<0.001
Reader 2	4.63 (2.02)	5.58 (2.17)	7.12 (2.76)	<0.001 ‡	<0.001	<0.001	<0.001

Note: ICC = intraclass correlation coefficient. ASIR-V 50% = adaptive statistical iterative reconstruction-Veo at a level of 50%; DLIR-M and DLIR-H = deep learning image reconstruction in medium and high strengths, respectively. † indicates normally distributed data compared using repeated measures ANOVA. ‡ indicates nonnormally distributed data compared with the Friedman test.

**Table 4:** Comparison of CNR among the three groups.

CNR	ASIR-V 50%	DLIR-M	DLIR-H	*p*	*p*		
					ASIR-V 50% vs. DLIR-M	ASIR-V 50% vs. DLIR-H	DLIR-M vs. DLIR-H
Aorta							
ICC	0.953	0.948	0.938				
Reader 1	4220.40 (1543.54)	7023.38 (3140.01)	15,958.01 (6466.08)	<0.001 ‡	<0.001	<0.001	<0.001
Reader 2	4234.37 (1612.33)	7126.66 (3468.47)	15,880.75 (6711.26)	<0.001 ‡	<0.001	<0.001	<0.001
Lung							
ICC	0.869	0.893	0.924				
Reader 1	41.75 (19.53)	48.10 (22.55)	57.13 (33.65)	<0.001 ‡	<0.001	<0.001	<0.001
Reader 2	42.78 (21.31)	49.55 (25.71)	57.05 (34.88)	<0.001 ‡	0.009	<0.001	<0.001
Muscle							
ICC	0.948	0.932	0.900				
Reader 1	3239.26 (888.57)	5560.85 (1350.57)	11,430.93 (3465.19)	<0.001 ‡	<0.001	<0.001	<0.001
Reader 2	3202.04 (1440.15)	5533.11 (1680.58)	11,239.74 (3792.47)	<0.001 ‡	<0.001	<0.001	<0.001
Liver							
ICC	0.926	0.864	0.862				
Reader 1	3353.37 ± 648.20	5673.28 ± 1184.25	12,621.82 ± 2547.01	<0.001 †	<0.001	<0.001	<0.001
Reader 2	3343.94 ± 620.62	5624.45 ± 1051.58	12,459.73 ± 2273.57	<0.001 †	<0.001	<0.001	<0.001
Vertebrae							
ICC	0.921	0.833	0.791				
Reader 1	1716.03 (757.72)	2641.56 (895.71)	4391.59 (1575.93)	<0.001 ‡	<0.001	<0.001	<0.001
Reader 2	1797.66 (641.32)	2618.25 (916.38)	4304.24 (1915.51)	<0.001 ‡	<0.001	<0.001	<0.001

Note: ICC = intraclass correlation coefficient. ASIR-V 50% = adaptive statistical iterative reconstruction-Veo at a level of 50%; DLIR-M and DLIR-H = deep learning image reconstruction in medium and high strengths, respectively. † indicates normally distributed data compared using repeated measures ANOVA. ‡ indicates nonnormally distributed data compared with the Friedman test.

**Table 5:** Results of the subjective image analysis.

Subjective Scores	ASIR-V 50%	DLIR-M	DLIR-H	*p*
Soft tissue				
Reader 1	3 (0)	4 (0)	5 (0)	<0.001
Reader 2	3 (0)	4 (0)	5 (0)	<0.001
Lung tissue				
Reader 1	4 (0)	4 (0)	4 (0)	0.121
Reader 2	4 (0)	4 (0)	4 (0)	0.069

Note: ASIR-V 50% = adaptive statistical iterative reconstruction-Veo at a level of 50%; DLIR-M and DLIR-H = deep learning image reconstruction at medium and high strengths, respectively.

## Data Availability

The data presented in this study are available on request from the corresponding author. The data are not publicly available due to privacy or ethical concerns.
